# Impaired SARS-CoV-2 specific T-cell response in patients with severe COVID-19

**DOI:** 10.3389/fimmu.2023.1046639

**Published:** 2023-04-17

**Authors:** Lidewij W. Rümke, Wouter L. Smit, Ailko Bossink, Gijs J. M. Limonard, Danya Muilwijk, Lenneke E. M. Haas, Chantal Reusken, Sanne van der Wal, Bing J. Thio, Yvonne M. G. van Os, Hendrik Gremmels, Jeffrey M. Beekman, Monique Nijhuis, Annemarie M. J. Wensing, Michiel Heron, Steven F. T. Thijsen

**Affiliations:** ^1^ Department of Medical Microbiology and Immunology, Diakonessenhuis Utrecht, Utrecht, Netherlands; ^2^ Virology, Department of Medical Microbiology, University Medical Center Utrecht, Utrecht, Netherlands; ^3^ Department of Pulmonary Diseases, Diakonessenhuis Utrecht, Utrecht, Netherlands; ^4^ Department of Pediatric Pulmonology, Wilhelmina Children’s Hospital, University Medical Center, Utrecht University, Utrecht, Netherlands; ^5^ Regenerative Medicine Center Utrecht, University Medical Center, Utrecht University, Utrecht, Netherlands; ^6^ Department of Intensive Care, Diakonessenhuis Utrecht, Utrecht, Netherlands; ^7^ Centre for Infectious Disease Control, WHO Reference Laboratory for COVID-19, National Institute for Public Health and the Environment (RIVM), Bilthoven, Netherlands; ^8^ Occupational Health Office, Department of Human Resources, University Medical Center Utrecht, Utrecht, Netherlands

**Keywords:** SARS-CoV-2, COVID-19 ELISpot IFN-γ release assay, T-cell immunity, spike protein, nucleocapsid protein, membrane protein

## Abstract

Cellular immune responses are of pivotal importance to understand SARS-CoV-2 pathogenicity. Using an enzyme-linked immunosorbent spot (ELISpot) interferon-γ release assay with wild-type spike, membrane and nucleocapsid peptide pools, we longitudinally characterized functional SARS-CoV-2 specific T-cell responses in a cohort of patients with mild, moderate and severe COVID-19. All patients were included before emergence of the Omicron (B.1.1.529) variant. Our most important finding was an impaired development of early IFN-γ-secreting virus-specific T-cells in severe patients compared to patients with moderate disease, indicating that absence of virus-specific cellular responses in the acute phase may act as a prognostic factor for severe disease. Remarkably, in addition to reactivity against the spike protein, a substantial proportion of the SARS-CoV-2 specific T-cell response was directed against the conserved membrane protein. This may be relevant for diagnostics and vaccine design, especially considering new variants with heavily mutated spike proteins. Our data further strengthen the hypothesis that dysregulated adaptive immunity plays a central role in COVID-19 immunopathogenesis.

## Introduction

Since December 2019, coronavirus disease 2019 (COVID-19) cases have exceeded 550 million and resulted in more than 6 million deaths worldwide despite global control measures (1). Infections with SARS-CoV-2 show a broad clinical severity spectrum ranging from asymptomatic infection to life-threatening COVID-19. Important hallmarks of severe disease are misdirected immune responses with ongoing cytokine production, profound lymphopenia and skewed T-cell populations (2–4). T-lymphocytes (T-cells) have a prominent role in the early control and clearance of the virus. Characterization of T-cell response kinetics in relation to clinical phenotypes helps to understand disease progression, which is key for identification of drug and vaccine targets. Most studies have focused on general changes in the number and functionality of all peripheral blood T-cells in the acute phase of severe COVID-19, whereas few studies measured dynamics of SARS-CoV-2 specific T-cells using *in vitro* activation assays in large cohorts of individuals with different disease severity in both the acute and convalescent phase (4–11). In this study, we longitudinally characterized SARS-CoV-2 specific T-cell responses to the structural spike, membrane and nucleocapsid proteins with an enzyme-linked immunosorbent spot (ELISpot) interferon-γ release assay in patients with mild, moderate, and severe COVID-19 to improve understanding of COVID-19 immunopathogenesis.

## Materials and methods

### Ethics statement

Samples of hospitalized patients were collected as part of a study approved by the Medical Research Ethics Committees United (Nieuwegein, the Netherlands; MEC-U: NL73618.100.20). In addition, samples from a health care workers study approved by the UMCU Institutional Review Board (ABR NL73903.041.20) were used. Written informed consent was obtained from all enrolled clinical patients and health care workers.

### Study participants and sample collection

Two cohorts of SARS-CoV-2 infected subjects were included: hospitalized COVID-19 patients (n=190) and non-hospitalized SARS-CoV-2 infected health care workers (n=58). Mild disease was defined as asymptomatic or symptomatic infection without need for hospitalization, moderate disease as infection requiring hospitalization and severe disease as infection meeting the definition of moderate disease while also requiring admission to the intensive care unit (ICU), invasive mechanical ventilation or occurrence of death during admission. For a division of the cohort based on fatal clinical outcome (deceased during hospitalization), not all patient data could be retrieved, allowing us to analyze 164 of the 190 patients.

COVID-19 diagnosis was confirmed by reverse transcriptase polymerase chain reaction (RT-PCR) on combined nasopharyngeal swabs. Hospitalized patients were recruited between August 2020 and December 2021 at the Diakonessenhuis hospital Utrecht, the Netherlands. Based on national surveillance data, dominant SARS-CoV-2 strains throughout the study period were the original Wuhan (Wuhan-Hu-1) strain, the Alpha (B.1.1.7) variant (emergence in December 2020) and the Delta (B.1.617.2) variant (emergence from June 2021). All patients were included before emergence of the Omicron (B.1.1.529) variant (12). Blood samples were collected at enrolment (within 3 days after hospital admission), 14 days after enrolment or at discharge from the hospital and up to 6 months after discharge. Subjects with mild disease were recruited between January and July 2021 and consisted of health care workers of the University Medical Center Utrecht, the Netherlands. Blood samples of health care workers were collected within the first week after symptom onset and three weeks later. Clinical information (demographics, comorbidities, vaccine status, day of symptom onset, disease severity) was obtained from the electronic medical record and in non-hospitalized patients *via* electronic daily questionnaires during 3-week follow-up (Castor, v2021.6.5).

### Isolation of peripheral blood mononuclear cells

Peripheral blood mononuclear cells (PBMCs) were isolated from heparinized peripheral blood using a Ficoll density gradient within 24h after collection. PBMCs of patients with moderate and severe disease were used for ELISpot directly after isolation. PBMCs of health care workers with mild disease severity were stored at -150°C after isolation and thawed on the day the assay was performed.

### SARS-CoV-2 ELISpot assay

To measure SARS-CoV-2 specific T-cell reactivity, an in-house developed ELISpot assay was performed, similar to a previously described procedure except for the addition of spike, membrane and nucleocapsid wild-type SARS-CoV-2 peptide pools (13). Per sample, 6 wells of an ELISpot^PRO^ plate precoated with polyvinylidene difluoride (Mabtech, Nacka Strand) were used. In these wells, 100 µl of 2.5x10^6^ PBMCs/ml were stimulated with 50 µl of a mitogen control (anti-human CD3 monoclonal antibody CD3-2 [0.1 µg/ml], Mabtech), a negative control (AIM-V medium, Life Technologies, Invitrogen) and 4 PepTivator^®^SARS-CoV-2 lyophylized peptide pools, consisting of 15-mer sequences with 11 amino acids overlap: Prot_S (covering the immunodominant sequence domains of the spike glycoprotein), Prot_S1 (covering the N-terminal S1 domain of the S glycoprotein), Prot_M (covering the complete sequence of the membrane glycoprotein), or Prot_N (covering the complete sequence of the nucleocapsid phosphoprotein; GenBank MN908947.3; protein QHD43416.1, QHD43419.1, QHD43423.2, QHD43416.1). The number of SARS-CoV-2-specific interferon (IFN)-γ-secreting T-cells/2.5x10^5^ PBMCs were measured using an ELISpot Reader (Autoimmun Diagnostika GmbH). The spot forming cell (SFC) size was based on the expected SFC size of an IFN-γ-producing T-cell as determined by *Feske et al.* and was set on -2.8 log (mm^2^) (14). Ten or more SFC, induced by the spike, membrane and nucleocapsid peptide pools combined, was considered most indicative for COVID-19 disease (13), yielding the highest sensitivity and specificity in both mild/asymptomatic disease (15) and moderate/severe disease (13). The average number of SFC induced by the mitogen control was comparable for both fresh and thawed PBMCs.

The in-house assay was compared to the CE IVD Oxford Immunotec’ research-use only (RUO) T-SPOT.COVID test which uses a standardized ELISpot based technique (Package Insert: T-SPOT.COVID-PI-UK-000, Revision number: 3 Date of Issue: 26 February 2021). Twenty-four blood samples, collected from 8 SARS-CoV-2 seronegative health care workers and 16 COVID-19 patients, were tested simultaneously with both the in-house developed SARS-CoV-2 ELISpot and the T-SPOT.COVID test. The number of days after symptom onset of the COVID-19 patients varied between 49 and 159 days. At the time of sampling, 10 out of 16 COVID-19 patients and all of the seronegative HCW had not been vaccinated. Two of these vaccinated COVID-19 patients had received the first shot and 4 were fully vaccinated.

### Flow cytometry

Flowcytometric analysis of lymphocyte subsets was performed by using the BD FACSCanto II flowcytometer (Becton Dickinson Life Sciences). Blood samples were kept at room temperature and analyzed upon arrival within 48 hours after collection. For the identification and enumeration of T-cells (CD3+, CD3+CD4+, CD3+CD8+), B-cells (CD3-CD19+) and NK-cells (CD3-CD16/CD56+) within leukocytes (CD45+), two monoclonal antibody reagent panels were used. A BD Trucount tube, consisting of CD45-PerCP/CD4-APC/CD8-PE/CD3-FITC four-color monoclonal antibody cocktail and a second BD Trucount tube consisting of CD45-PerCP/(CD56 + CD16)-PE/CD19-APC/CD3-FITC. The assays were performed according to the manufacturer’s protocol.

### Statistical analysis

Categorical variables were described as frequencies and percentages and compared using the Fisher’s exact test. Continuous variables were expressed as median with interquartile ranges (IQR) or mean with standard deviation (SD), and compared using the independent t-test, Mann-Whitney U test or Kruskall Wallis test, depending on the distribution of the variables. The in-house developed ELISpot and the commercial T-SPOT.COVID test were compared using Bland-Altman analysis and t-test statistics. Flow cytometry data was used to calculate the number of T-lymphocytes loaded in the ELISpot assay as follows: (percentage of lymphocytes) x (250.000 PBMCs) x (CD3/(CD3 + CD16.56 + CD19)). Longitudinal trends of SARS-CoV-2 specific T-cell responses (in SFC count data) in moderate and severe COVID-19 patients can be analyzed using Poisson-type regression models. We used a negative binomial mixed model, which are robust models to statistically analyze longitudinal count data (16). The number of SFC of the combined and separate peptide pools (spike, membrane, nucleocapsid) were defined as outcome variables in separate models. Each model included a random intercept per subject and fixed effects for days after symptom onset, disease severity group (moderate and severe) and the interaction between days after symptom onset and disease severity group. Coefficients with 95% confidence intervals are generated as a log-link to the outcome (natural log). A natural spline with 3 degrees of freedom for days after symptom onset was included to account for non-linearity of the SARS-CoV-2 specific T-cell response curves. The likelihood ratio test was used to compare model fit of a model with and without the interaction term for days after symptom onset and disease severity group, to assess whether the response curves significantly differed between groups. To test whether the response curves significantly differed between groups, a likelihood ratio test was used to compare models with and without the interaction term between days after symptom onset and disease severity group. *P-*values less than 0.05 were considered statistically significant. Statistical analyses were performed using SPSS software (version 26 for Windows; Chicago, Illinois, USA) and R (version 4.1.1). Figures were made using R and GraphPad Prism (version 9.3.0).

## Results

### Dynamics of SARS-CoV-2 specific T-cell responses in hospitalized COVID-19 patients

90 patients with moderate and 100 patients with severe COVID-19 were included. Severe COVID-19 was defined as disease which prompted admission to the intensive care unit (ICU), invasive mechanical ventilation or which led to occurrence of death during hospital admission. Patients’ characteristics are presented in **Table 1**. A total of 390 samples (observations) was obtained; two or more sequential samples were available from 63 patients with moderate and 21 patients with severe COVID-19. Sixty-one patients were sampled up to 1 to 6 months after symptom onset. Antigen-specific T-cell immunity was measured using *in vitro* stimulation of PBMCs with SARS-CoV-2 spike, membrane and nucleocapsid peptide pools. At hospital admission, median SFC counts induced by these peptide pools were 38 SFC (IQR 13-124) in moderate vs 16 SFC (IQR 5-55) in severe COVID-19 patients (Mann-Whitney *U* = 2876, *p*<.001), compared to 1 SFC in seronegative controls. The median symptom duration at hospital admission did not differ between both severity groups (12 [IQR 9-14] days in moderate and 13 [IQR 9-16] days in severe patients, *p*=.174).

**Table 1:** Baseline characteristics of hospitalized patients with moderate or severe COVID-19.

	Total	Moderate	Severe	*p*-value
	n=190	n=90	n=100	
Age (median [IQR])	65 [55, 73]	65 [53, 73]	65 [56, 73]	0.377
Female gender (%)	75 (39)	34 (38)	41 (41)	0.659
BMI (median [IQR])	28.1 [24.9, 32.6]	26.8 [24.4, 32.7]	29.7 [25.3, 32.3]	0.184
Current smoker (%)	36/108 (33)	29/65 (45)	7/43 (15)	0.003
Medical history				
Hypertension (%)	72/174 (41)	25/90 (28)	47/84 (57)	<0.001
Cardiovascular disease (%)	54/169 (32)	26/90 (29)	28/79 (35)	0.410
Chronic pulmonary disease (%)	54/168 (32)	26/90 (29)	28/78 (36)	0.408
Diabetes mellitus (%)	49/172 (28)	22/90 (24)	27/82 (33)	0.240
Medication				
Pre-existent immunomodulating drugs (%)	19/163 (12)	6/90 (7)	13/73 (18)	0.047
Steroids during follow-up (%)	149/164 (91)	77/90 (86)	72/74 (97)	0.012
Vaccinated at first sampling*				
Yes (%)	3 (2)	0	3 (3)	N/A
No (%)	132 (69)	87 (97)	45 (45)	N/A
Unknown (%)	55 (29)	3 (3)	52 (52)	N/A
Symptom duration at first sampling (days; median [IQR])	12 [9, 15]	12 [9, 14]	13 [9,16]	0.174
Disease course				
ICU admission (%)	N/A	0	67/76 (88)	N/A
Intubation (%)	N/A	0	51/73 (70)	N/A
Death (%)	N/A	0	29/74 (39)	N/A

Values are expressed as median with interquartile ranges or n (%). P-values were calculated using the Fisher’s exact (categorical data) or Mann-Whitney U test (continuous variables with a non-normal distribution); N/A, not applicable; BMI, Body mass index; ICU, Intensive Care Unit. *Either partially or fully vaccinated with any SARS-CoV-2 vaccine.

**Figure 1:**
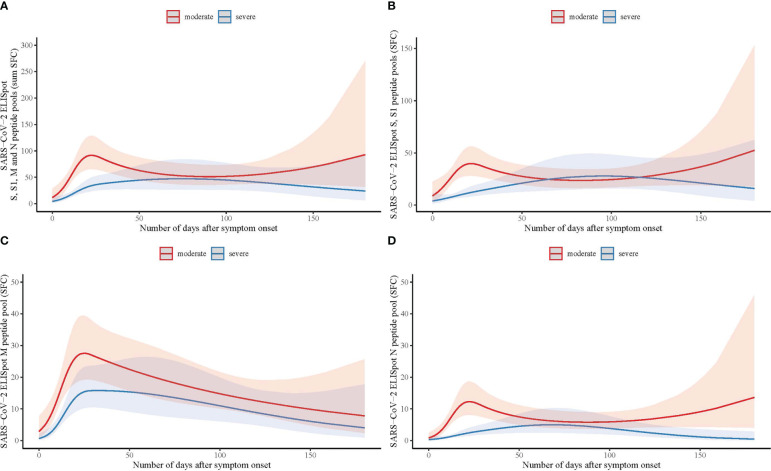
Longitudinal analysis of SARS-CoV-2 specific T-cell responses measured by ELISpot with spike, membrane and nucleocapsid peptide pools in hospitalized patients with moderate and severe COVID-19. **(A)** Number of days after symptom onset in relation to T-cell reactivity with the sum of the total number of spot forming cells (SFC) measured by ELISpot with spike (S and S1), membrane (M), and nucleocapsid (N) peptide pools in moderate (n=90; red line) and severe COVID-19 (n=100; blue line) patients, using a binominal mixed model with the representation of the confidence limits by the shaded areas. **(B)** As **(A)** with the number of SFC measured with the S and S1 peptide pools. **(C)** As **(A)** with the number of SFC measured with the M peptide pool. **(D)** As **(A)** with the number of SFC measured with the N peptide pool.

Longitudinal trends of SARS-CoV-2 specific T-cell responses curves were different between patients with moderate and severe COVID-19 disease (likelihood ratio test *p*=.037; **Figure 1A**; **Supplementary Figure 1A**; **Supplementary Table 1**), with an impaired SARS-CoV-2 specific T-cell response in patients with severe disease relative to moderate disease. The difference in response curves between moderate and severe disease was most apparent in the first 30 days after symptom onset (**Figure 1**). Analyses of the separate peptide pools showed that these differences were also present for spike (S-protein) and nucleocapsid (N-protein) specifically, whilst there was no significant difference for membrane protein (M-protein)-specific responses (spike *p*=.001; membrane *p*=.336; nucleocapsid *p*=.015; **Figures 1B–D** and **Supplementary Figures 1B–D**).

Immunophenotyping using flow cytometry on a subset of PBMCs collected at hospital admission of moderate (n=50) and severe (n=37) patients was used to calculate the total number of T-lymphocytes (CD3+) loaded in the ELISpot assay. The total number of T-lymphocytes did not differ in moderate and severe patients: 110417 ± 42035 vs 100804 ± 36984, *p*=.271), suggesting that the difference in antigen-specific T-cell counts between both groups was not attributed to differences in the total number of T-lymphocytes in the peripheral blood of the patients.

In addition to a subanalysis based on disease severity, we looked at T-cell responses in patients with fatal disease, who deceased during hospitalization, and those who recovered from COVID-19. Interestingly, at the moment of hospitalization, we found a significantly lower T-cell response directed against peptides from S-protein, N-protein and M-protein, in patients who had a fatal disease course (**Supplementary Figure S2**). A low or absent (< 10 SFC) response against the N- and M- protein in particular, was a predictor of fatal COVID-19. Patients with fatal disease were on average 10 years older (*p*=.001) and suffered more frequently from pulmonary and hypertensive comorbidity (*p*=.005 and *p*=.046 respectively) (**Supplementary Table 2**). There was no correlation between age and median T-cell response to any of the peptide pools (Spearman correlation).

### SARS-CoV-2 specific T-cell responses in non-hospitalized COVID-19 patients

**Figure 2:**
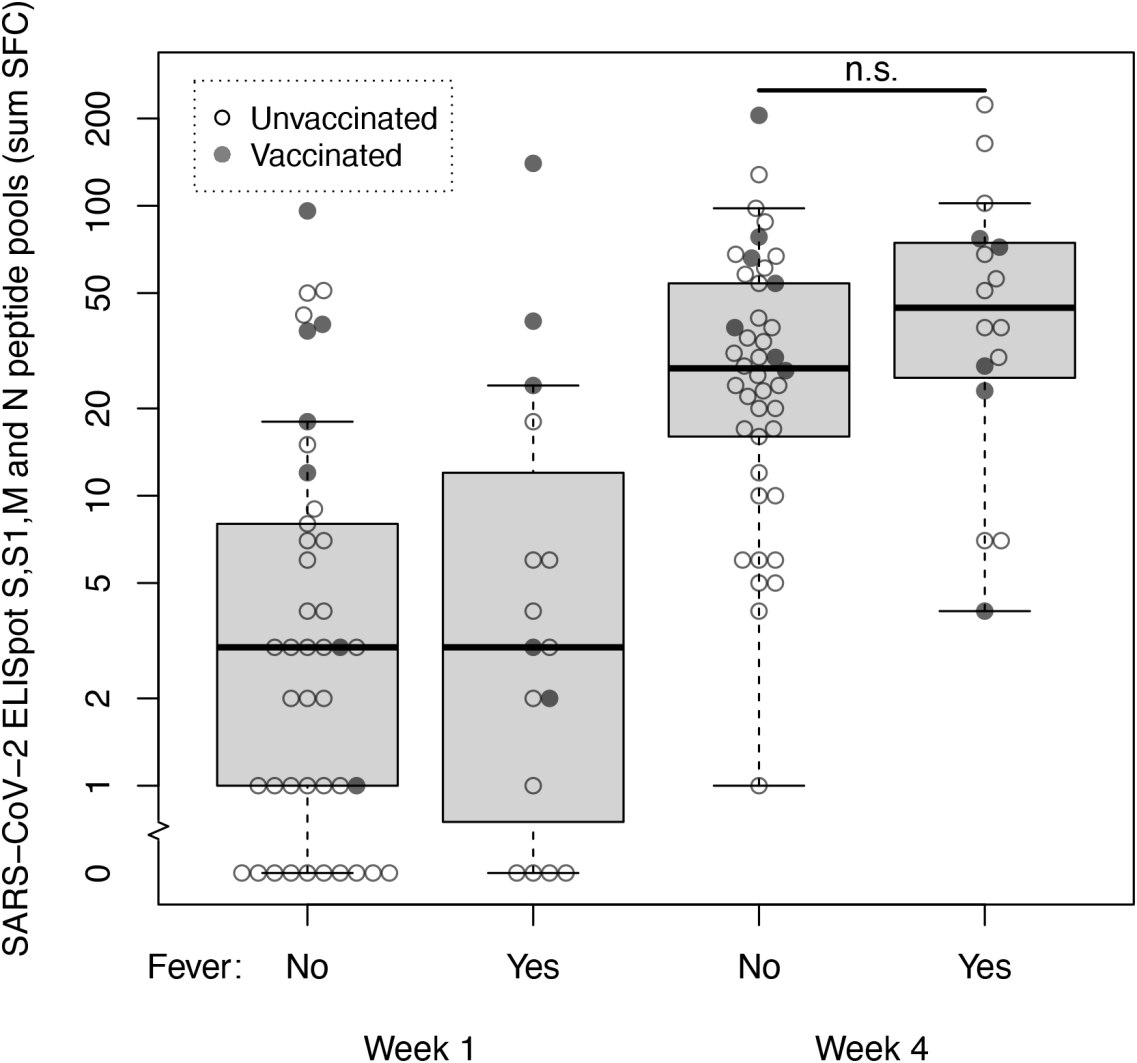
SARS-CoV-2 specific T-cell responses at week 1 and 4 after symptom onset measured by ELISpot with spike, membrane and nucleocapsid peptide pools in non-hospitalized subjects with mild COVID-19. T-cell reactivity against spike (S and S1), membrane (M) and nucleocapsid (N) peptide pools (sum SFC) measured by ELISpot in week 1 and 4 after symptom onset in healthcare workers with mild COVID-19 without fever (n=42) and with fever (n=16). Open dots represent unvaccinated subjects, closed dots represent subjects who were either fully or partially vaccinated with any SARS-CoV-2 vaccine before sample collection. Box and whiskers represent median, interquartile, minimum and maximum values; n.s., not significant.

We then explored SARS-CoV-2 specific T-cell responses in a cohort of 58 non-hospitalized health care workers with mild COVID-19 (**Supplementary Table 3**). Using a cut-off of 10 SFC, specific SARS-CoV-2 T-cells immunity was present in 13 of 58 (22%) subjects in the first week after symptom onset. Eight of these 13 subjects were either partially or fully vaccinated. In the fourth week after symptom onset specific SARS-CoV-2 T-cell immunity was present in 47 of 58 (81%) subjects. Median SFC counts at these time points were 3 SFC (IQR 1-8) in week 1 vs 30 SFC (IQR 14-60) in week 4. Sixteen of 58 individuals reported fever (≥ 38°C) at any time point during follow-up, which we considered a marker for disease severity (17). At week 4, median SFC counts in subjects with fever vs subjects without fever were 45 SFC (IQR 26-75) vs 28 SFC (IQR 16-54); the median SFC counts of these two groups did not differ significantly (Mann-Whitney *U* = 244, *p*=.109) (**Figure 2**). When spike and non-spike peptide pools were analyzed separately, also no significant difference at week 4 was found in spike SFC in subjects with fever vs subjects without fever (24 SFC [IQR 15-36] vs 13 SFC [IQR 5-26]; Mann-Whitney *U =* 306, *p*=.587) or the combined membrane and nucleocapsid SFC (22 SFC [IQR 7-49] vs 14 SFC [IQR 7-26]; Mann-Whitney *U =* 321, *p*=.784).

### Contribution of spike, membrane and nucleocapsid peptide pools to SARS-CoV-2 specific T-cell responses

**Figure 3:**
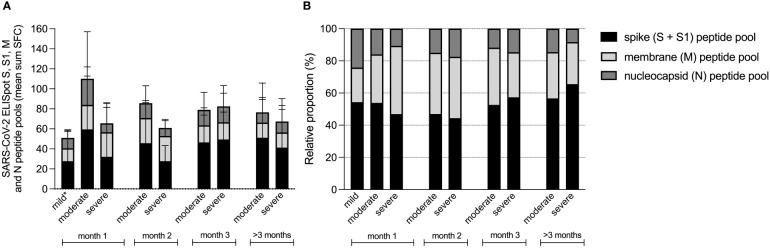
Contribution of spike, membrane and nucleocapsid peptide pools to the SARS-CoV-2 specific T-cell response in patients with mild, moderate and COVID-19. Absolute **(A)**, and relative **(B)** proportion presented as mean percentage of spike (S and S1), membrane (M) and nucleocapsid (N) peptide pools of total SARS-CoV-2 specific T-cell reactivity (sum SFC of S, S1, M and N). Data derived from sera that were collected per month after symptom onset, measured by ELISpot in patients with mild (n=58), moderate (n=90) and severe COVID-19 (n=100). *T-cell responses from mild disease group were obtained from thawn PBMCs, whereas the T-cell responses from hospitalized patient with moderate or severe disease were measured in fresh PBMCs. Comparability was ensured based on the presence of a similar mitogen response between both sample groups.

Next, we investigated the absolute and relative contributions of the separate peptide pools of total SARS-CoV-2 specific T-cell reactivity (sum of SFC) in samples collected 14-28 days after symptom onset (mild n=48, moderate n=36, severe n=30). First, it must be noted that individuals with mild disease elicited only a weak T-cell response (combined N-, M- and S-protein specific T-cell responses) in quantitative terms (**Figure 3A**). It is important to emphasize that samples in this group were processed differently (thawn PBMCs) compared to the two hospitalized cohorts (fresh PBMCs) although the magnitude of the mitogen control response was similar between all groups, allowing for comparability. Also, individuals with mild disease were sampled relatively early during their disease course compared to hospitalized patients. As already shown in **Figure 1**, T-cell responses during the first month were lower in severe compared to moderate disease, a difference that disappeared over time.

To compare the magnitude of INF-γ-secreting antigen-specific T-cell responses within each group, we then assessed the relative contributions by comparing the relative proportion of each antigen-specific response (spike response comprised the combined response to S1 + S2 peptide pools) to the cumulative response (**Figure 3B**). Here we found that the highest proportions were induced by the spike peptide pools (mean percentage 47% ± 20% of total SFC) and the membrane protein peptide pool (33% ± 22% of total SFC), whereas the nucleocapsid peptide pool resembled the smallest proportion (20% ± 19% of total SFC). When these relative proportions per disease severity group were compared, the proportion of spike protein induced T-cell responses did not differ between the three groups (49 ± 17% of total SFC in mild, 48 ± 21% of total SFC in moderate and 42 ± 25% of total SFC in severe patients (Kruskall Wallis test, H(2)=3.141, *p*=.208). During the first month of the infection, membrane protein induced T-cell responses comprised the largest proportion of the virus-specific T-cell response in severe cases (25% ± 16% of total SFC in mild, 31% ± 16% of total SFC in moderate and 48% ± 28% of total SFC in severe; H(2)=14.991, *p*=.001), whereas nucleocapsid induced T-cell responses represented the smallest proportion in severe cases (25 ± 18% of total SFC in mild, 21 ± 19% of total SFC in moderate and 10 ± 19% of total SFC in severe; H(2)=24.579, *p*=.000).

During follow-up of the moderate and severe COVID-19 patients, relative contributions of the membrane protein induced T-cell response decreased.

### Performance in-house SARS-CoV-2 ELISpot test

Finally, we compared the in-house developed SARS-CoV-2 ELISpot to the RUO version of the T-SPOT.COVID test. The qualitative interpretation of T-cell reactivity, assessed according to the manufacturers’ instructions for the T-SPOT.COVID test and using a cut-off of 10 SFC for the in-house ELISpot showed 100% concordance. **Supplementary Figure 3A** shows the results of the T-cell reactivity against the spike (S1), membrane and nucleocapsid peptide pools (Spearman’s rho: 0.92 95% CI:0.88 - 0.95), *p*<.001). Bland-Altman analysis showed no significant bias between both assays comparing the results against the individual peptides (mean difference: -1.27% (95% CI: -30.9 - 28.3), **Supplementary Figure 3B**). None of the SARS-CoV-2 seronegative individuals showed more than 1 SFC against any peptide in both assays. The combined T-cell reactivity in COVID-19 patients, represented by the sum of spike S1, membrane and nucleocapsid SFC, did not differ between the in-house SARS-CoV-2 ELISpot and the T-SPOT.COVID test (82 SFC [IQR 50-113] vs 84 SFC [IQR 36-106], *p*=.92) (**Supplementary Figure 3C**).

## Discussion

Longitudinal analysis of functional cellular immune responses using ELISpot reveals impaired SARS-CoV-2 specific T-cell reactivity in severe COVID-19 patients compared to hospitalized patients with moderate disease. Furthermore, we show that a low T-cell response at the moment of hospitalization is indicative of a fatal disease course. These findings support the hypothesis that dysregulated T-cell responses in the early phase of infection play a central role in COVID-19 immunopathogenesis and might serve as a useful prognostic marker for disease progression and immune directed treatment upon hospital admission.

Our data is in line with several studies reporting lower virus-specific T-cell responses in patients with severe disease and early induction of virus-specific T-cells in mild cases (8, 10, 18–22). Some studies showed that the quantity of SARS-CoV-2–specific T-cells was not proportional to disease severity (8, 10, 23), or that the impaired virus-specific T-cells response is detected in CD4+ lymphocytes of severe patients in the acute phase of infection, but not in CD8+ lymphocytes (11). Furthermore, a few reported an equal or even higher virus-specific T-cell reactivity in severe or critical cases in the convalescent phase of infection (5, 24, 25). It is important to note that time of sampling influences the magnitude of detected virus-specific T-cell responses, supported by our longitudinal analysis showing a significantly decreased response in the early phase of severe infection but no differences in the convalescent phase. Also, different techniques to measure SARS-CoV-2 specific T-cells, e.g. ELISpot, activation induced markers (AIM) assays and intracellular cytokine stainings (ICS), as well as the peptides that are used in the assays might accentuate different parts of the T-cell immune response.

Different hypotheses for the decreased T-cell response in the acute phase of severe COVID-19 include sequestration to the respiratory tract (6, 18, 26), apoptosis of T-cells by direct viral infection (27), functional impairment and exhaustion of T-cells (18, 21, 28), impaired levels and function of dendritic cells (29), and a defective induction of virus-specific T-cells (10). Concerning virus-specific regulatory T-cells, there have been contradictory reports of either decreased or increased proportions of these cells, possibly affecting differentiation of virus-specific effector T-cells (21, 26). In addition to cellular immunity, antibodies are an important arm of adaptive immunity. Interestingly, antibodies against SARS-CoV-2 seem to be generated rapidly and abundantly independent of disease severity, indicating that a robust virus-specific antibody response alone is not enough to prevent progression to severe disease (6, 18, 29). In our cohort of hospitalized COVID-19 patients and that of others, a heterologous B-cell response could be shown due to cross-reactivity directed at the spike protein (30, 31). These responses were associated with severe disease, and could be explained by homologous epitopes in the conserved S2 domain of the spike protein of seasonal/endemic Betacoronaviruses and the novel SARS-CoV-2 virus. Thus, although an unfavorable disease course is characterized by a quantitatively robust humoral response in most cases, the quality of this response, in terms of breadth and potency, to control the infection could still be compromised (32). Furthermore, the presence of certain antibodies is associated with more severe clinical outcomes (33), e.g. type 1 IFN (IFN-1) autoantibodies causing diminished IFN-1 activity (34). These autoantibodies also affect T-cell expansion and differentiation, which depends on IFN-1 activation (35). Taken together, an early and balanced response of both B- and T-cells seems essential to curb viral replication, which in turn may dampen ongoing immune stimulation and disease severity.

In all study subjects a substantial part of the SARS-CoV-2 specific T-cell response was directed against the membrane protein, with the highest proportions found during the early phase (first month) of severe disease. This is in line with a previous report, that demonstrated predominance of membrane protein specific T-cell responses in severe cases (36). This response decreased over time (37). Hypothetically, it may reflect skewed immunity towards conserved membrane protein derived T-cell epitopes that is less effective and thus counterproductive, or a state of hyperinflammation in severe disease with skewing towards immunodominant epitope regions on the membrane protein. In-depth epitope screening of INF-γ secreting T-cell populations is warranted to further explore these hypotheses.

In contrast to our in-house ELISpot, the CE IVD version of the T-SPOT.COVID test lacks the membrane protein as antigenic stimulant. As most known mutations have occurred in the spike protein, the detection of T-cell reactivity against new SARS-CoV-2 variants may depend on stimulation with other structural SARS-CoV-2 proteins (38). Disregarding T-cell reactivity against SARS-CoV-2 membrane protein may decrease the detection rate of (past) SARS-CoV-2 infection, in particular concerning new variants with heavily mutated spike proteins. This finding has important implications for diagnostics as well as vaccine development and immunity against future SARS-CoV-2 variants of concern.

Our study provides important insights into the dynamics of the SARS-CoV-2 cellular immune response in COVID-19 patients with different disease severity. However, the study design has a few limitations that ought to be mentioned. First of all, alternative processing of PBMCs from patients with mild disease, which were frozen and later thawed instead of used directly after isolation as hospitalized patients, may have affected T-cell immunogenicity and skewing of the response (39). However, since the mitogen response did not differ significantly, we included this group in the comparison and showed both absolute and relative responses. The sampling time based on days after symptom onset was also different in patients with mild disease due to logistic reasons (hospitalized patients presented later, as it takes time to develop severe disease), which may explain the low T-cell response observed in the mild disease cohort. In addition, SARS-CoV-2 vaccination status was not known of all hospitalized patients, hampering statistical correction of spike T-cell responses induced by vaccination. However, due to late roll out of the Dutch vaccination campaign, and the fact that patients were advised against vaccination at least 3 months after infection, we expect very few vaccine effects present in the analysis. Moreover, a retrospective anamnestic screen in ~30 recovered patients by telephone revealed only two vaccinations that were received prior to hospitalization. Also, sensitivity analyses of the non-vaccine induced T-cell response directed at the nucleocapsid proteins, shows that the impaired response in severe compared to moderate COVID-19 patients remains present. Another limitation of our analyses is the fact that we did not correct for absence of follow-up samples of deceased patients and the use of T-cell suppressing medication (e.g. corticosteroids). The mitogen control was not affected in patients that received T-cell suppressing medication, indicating that the T-cell response endured under immunosuppressive treatment. At last, although highly sensitive, our ELISpot test is a single functional assay measuring IFN-γ producing cells, which does not allow assessment of the integrative immune response, such as activation state of other immune cells (interleukins, tumor necrosis factor alpha, PD-1) or discrimination between antigen-specific CD4+ and CD8+ T-cell responses. The ELISpot technique does measure both antigen presentation and T-cell activation. We are currently exploring the use of ELISpot supernatant to functionally measure other immune markers. This would be of interest to further enhance understanding of the underlying mechanisms of insufficient adaptive immune responses.

In summary, our study provides a comprehensive examination of longitudinal T-cell responses in a large number of patients with mild, moderate and severe COVID-19. We provide evidence of an impaired IFN-γ-secreting SARS-CoV-2-specific T-cell response during the initial phase of COVID-19 in patients with severe disease. This supports the hypothesis that optimal coordinated cellular immune responses drive viral clearance and limit disease severity and indicates that the presence of virus-specific cellular responses is an important prognostic factor for a favorable disease course and clinical outcome in hospitalized patients.

## Data availability statement

The original contributions presented in the study are included in the article/**Supplementary Material**. Further inquiries can be directed to the corresponding author.

## Ethics statement

The studies involving human participants were reviewed and approved by Medical Research Ethics Committees United (MEC-U), Nieuwegein, the Netherlands and UMCU Institutional Review Board. The patients/participants provided their written informed consent to participate in this study.

## Author contributions

LR contributed to patient recruitment, clinical data collection, data-analysis and wrote the manuscript; AB, GL, LH, BT, and YO contributed to patient recruitment and clinical data acquisition; SW performed experiments and data analysis; WS, HG, and DM performed data-analysis, interpretation of the data and revising the manuscript; CR, JB, MN, and AW contributed to study design, interpretation of the data and provided critical feedback; MH and ST contributed to study design, supervised the study and revised the manuscript. All authors contributed to the article and approved the submitted version.
