# Supplementary material for: Impaired SARS-CoV-2 specific T-cell response in patients with severe COVID-19
Source: Front Immunol. 2023 Apr 17;14:1046639. doi: 10.3389/fimmu.2023.1046639 (PMC10165493; doi:10.3389/fimmu.2023.1046639)
Supplement: Supplementary file 1 [file DataSheet_1.docx]

**Supplementary Material**

**Table S1. Negative binomial mixed effects model estimates of SARS-CoV-2 specific T-cell responses (in number of SFC) in moderate and severe COVID-19 patients.**

| n=190, number of observations=390 | Coefficient | 95% CI | Rate ratio | 95% CI | p-value |
| --- | --- | --- | --- | --- | --- |
| Intercept | 2.44 | 1.54 – 3.35 | 11.52 | 4.65 – 28.64 | <0.001 |
| Moderate disease | Reference group |  |  |  |  |
| Severe disease | -0.99 | -2.37 – 0.37 | 0.37 | 0.09 – 1.45 | 0.157 |
| Moderate disease : sp1(days after symptom onset) | 0.11 | -0.65 – 0.87 | 1.12 | 0.52 – 2.39 | 0.777 |
| Moderate disease : sp2(days after symptom onset) | 3.54 | 1.57 – 5.45 | 34.59 | 4.82 – 233.13 | <0.001 |
| Moderate disease : sp3(days after symptom onset) | 1.06 | -0.08 – 2.26 | 2.88 | 0.92 – 9.60 | 0.078 |
| Severe disease : sp1(days after symptom onset) | 1.82 | 0.41 – 3.27 | 6.18 | 1.51 – 26.28 | 0.012 |
| Severe disease : sp2(days after symptom onset) | 0.37 | -2.62 – 3.40 | 1.45 | 0.07 – 29.95 | 0.808 |
| Severe disease : sp3(days after symptom onset) | -0.24 | -2.09 – 1.62 | 0.79 | 0.12 – 5.04 | 0.799 |

Coefficients and 95% confidence intervals are on a natural log-scale. Rate ratios and accompanying 95% confidence intervals (CI) are calculated by exponentiation of the coefficients, so they can be interpreted on a normal scale. A natural spline with 3-degrees of freedom (sp) was used for days after symptom onset to model non-linearity of the number of spot forming cells (SFC) over time. The 3 different coefficients for the SFC count over time per disease severity group represent the 3 different components of the sp with 3-degrees of freedom. Model estimates are visualized in Figure 1A to support data interpretation.

**Table S2. Baseline characteristics of hospitalized non-fatal and fatal subjects with COVID-19**

|  | **Non-fatal n=135** | **Fatal n=29** | ***p*-value** |
| --- | --- | --- | --- |
| Age (median [IQR]) | 63 [53, 72] | 74 [67, 81] | <0.001 |
| Female gender (%) | 54/135 (40) | 11/29 (38) | 1.000 |
| BMI (median [IQR]) | 27.9 [24.9, 32.7] | 27.4 [24.4, 30.2] | 0.343 |
| Current smoker (%) | 34/93 (37) | 2/11 (18) | 0.322 |
| Hypertension (%) | 47/134 (35) | 15/26 (58) | 0.046 |
| Cardiovascular disease (%) | 35/133 (26) | 13/26 (50) | 0.021 |
| Chronic pulmonary disease (%) | 35/133 (26) | 15/27 (56) | 0.005 |
| Diabetes mellitus (%) | 32/134 (24) | 10/26 (38) | 0.145 |
| Preexistent immunomodulating drugs (%) | 12/133 (9) | 5/26 (19) | 0.159 |
| Steroids during followup (%) | 118/133 (89) | 26/26 (100) | 0.134 |
| ICU admission (%) | 43/134 (32) | 19/27 (70) | <0.001 |
| Intubation during hospitalisation (%) | 27/130 (21) | 19/27 (70) | <0.001 |

Values are expressed as median with interquartile ranges or n (%). *P*-values were calculated using the Fisher’s exact test (categorical data) or Mann-Whitney U test (continuous variables with a non-normal distribution); BMI, Body mass index; ICU, Intensive Care Unit.

**Table S3. Baseline characteristics of non-hospitalized subjects with mild COVID-19**

|  | **Total** | **No fever** | **Fever** | ***p*-value** |
| --- | --- | --- | --- | --- |
|  | **n=58** | **n=42** | **n=16** |  |
|  |  |  |  |  |
| Age (median [IQR]) | 33 [25, 45] | 29 [23, 38] | 45 [39, 54] | 0.001 |
| Female gender (%) | 49 (84) | 35 (83) | 14 (88) | 0.999 |
| BMI (median [IQR]) | 23.5 [21.9, 27.2] | 23.5 [21.6, 27.9] | 24.2 [22.8, 26.9] | 0.537 |
| Current smoker (%) | 3 (5) | 2 (5) | 1 (6) | 0.999 |
|  |  |  |  |  |
| *Medical history* |  |  |  |  |
| Cardiovascular disease (%) | 1 (2) | 1 (2) | 0 | N/A |
| Chronic pulmonary disease, including asthma (%) | 5 (9) | 4 (10) | 1 (6) | 0.999 |
| Diabetes mellitus | 0 | 0 | 0 | N/A |
|  |  |  |  |  |
| *Medication* |  |  |  |  |
| Pre-existent immunomodulating drugs (%) | 3 (5) | 3 (7) | 0 | N/A |
| Steroids during follow-up (%) | 2 (3) | 1 (2) | 1 (6) | 0.479 |
|  |  |  |  |  |
| *Vaccination status at inclusion* |  |  |  |  |
| Unvaccinated (%) | 46 (79) | 35 (83) | 11 (69) |  |
| Partially vaccinated (%) | 7 (12) | 4 (10) | 3 (19) | 0.469 |
| Fully vaccinated (%) | 5 (9) | 3 (7) | 2 (13) |  |
|  |  |  |  |  |

Values are expressed as median with interquartile ranges or n (%).
*P*-values were calculated using the Fisher’s exact test (categorical data)or Mann-Whitney U test (continuous variables with a non-normal distribution); N/A, not available; BMI, Body mass index.

**Figure S1. Longitudinal analysis of SARS-CoV-2 specific T-cell responses measured by ELISpot with spike, membrane and nucleocapsid peptide pools in hospitalized patients with moderate and severe COVID-19**


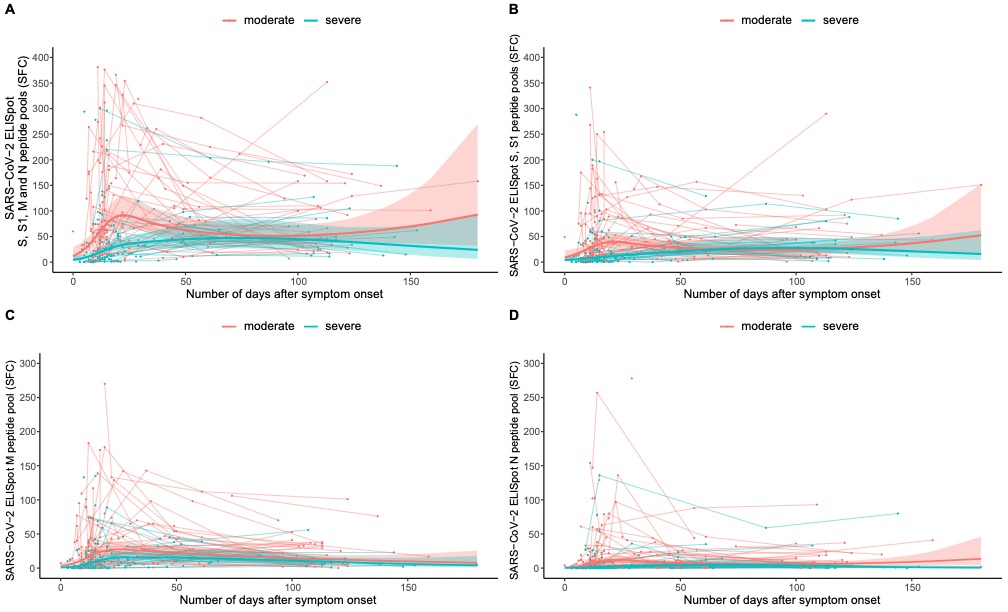


(A) Number of days after symptom onset in relation to T-cell reactivity with the sum of the total number of spot forming cells (SFC) measured by ELISpot with spike (S and S1), membrane (M), and nucleocapsid (N) peptide pools in moderate (n=90; red dots and connected lines) and severe COVID-19 (n=100; blue dots and connected lines) patients, using a binominal mixed model with the representation of the confidence limits by the shaded areas. (B) As (A) with the number of SFC measured with the S and S1 peptide pools. (C) As (A) with the number of SFC measured with the M peptide pool. (D) As (A) with the number of SFC measured with the N peptide pool.

**Figure S2. Comparison of T cell responses at hospital admission in patients with non-fatal and fatal disease**

(A) Boxplot analysis with inclusion of individual data points showing T-cell reactivity (SFC) against spike (S+S1), membrane (M) and nucleocapsid (N) peptide pools combined. (B) Boxplot analysis with inclusion of individual data points showing T-cell reactivity (SFC) against spike (S+S1) pools. (C) Boxplot analysis with inclusion of individual data points showing T-cell reactivity (SFC) against membrane (M) and nucleocapsid (N) peptide pools combined. Box and whiskers represent median, interquartile, minimum and maximum values.

**Figure S3. In-house SARS-CoV-2 ELISpot assay vs commercial T-SPOT.COVID test**

(A) Scatterplot showing T-cell reactivity (SFC) against individual spike (S1), membrane and nucleocapsid peptide pools simultaneously measured with T-SPOT.COVID test and in-house SARS-CoV-2 ELISpot. r = Spearman's rank correlation coefficient. (B) Bland-Altman plot presenting the same data as percentage difference of in-house ELISpot SFC compared with the average number of SFC per antigen by both assays, with the representation of the limits of agreement from -2SD to +2SD (dotted lines) and of confidence interval limits for the mean (shaded area). (C) T-cell reactivity against spike (S1), membrane and nucleocapsid peptide pools (sum SFC) simultaneously determined with T-SPOT.COVID test and in-house SARS-CoV-2 ELISpot in SARS-CoV-2 seronegative individuals (n=8) and in COVID-19 patients (n=16). Box and whiskers represent median, interquartile, minimum and maximum values; ns, not significant.
